# Assimilated Rank in the Navy

**Published:** 1850-09

**Authors:** Jno. S. Trowbridge


*Assimilated Rank in the Navy.*—At the semi-annual meeting of the Erie County Medical Society in the State of New York, held in the city of Buffalo, June 12th, 1850, it was,

On motion of Dr. Austin Flint,

*Resolved,* That this Society recommend to the members of the medical profession of this county, for their signatures, the memorial to Congress in behalf of the medical officers of the navy, praying for an assimilated rank, believing that the action on the part of our National Legislature asked for, is due, not only to the medical department of the navy, but to the character of the medical profession generally.

*Resolved,* That this resolution be published, and that copies be transmitted to the Representatives in Congress from this District, and to the Senators from this State. From the regular minutes,

Jno. S. Trowbridge, *Sec.*

